# Recent Development of Flax Fibres and Their Reinforced Composites Based on Different Polymeric Matrices

**DOI:** 10.3390/ma6115171

**Published:** 2013-11-12

**Authors:** Jinchun Zhu, Huijun Zhu, James Njuguna, Hrushikesh Abhyankar

**Affiliations:** 1 Centre of Automotive Technology, Cranfield University, Cranfield, MK43 0AL, UK;; 2 Cranfiled Health, Cranfield University, Cranfield, MK43 0AL, UK; E-Mail: h.zhu@cranfield.ac.uk; 3 Institute for Innovation, Design and Sustainability, Robert Gordon University, Aberdeen AB10 7GJ, UK; E-Mail: j.njuguna@rgu.ac.uk

**Keywords:** flax composites, mechanical properties, modifications

## Abstract

This work describes flax fibre reinforced polymeric composites with recent developments. The properties of flax fibres, as well as advanced fibre treatments such as mercerization, silane treatment, acylation, peroxide treatment and coatings for the enhancement of flax/matrix incompatibility are presented. The characteristic properties and characterizations of flax composites on various polymers including polypropylene (PP) and polylactic acid, epoxy, bio-epoxy and bio-phenolic resin are discussed. A brief overview is also given on the recent nanotechnology applied in flax composites.

## 1. Introduction

As a result of the growing environmental awareness (e.g., increased pollution, increasing demand for biodegradable materials, material need for CO_2_ neutrality and low greenhouse gas emissions, new environmental laws and regulations), manufacturers and scientists are keen to study novel environmental friendly materials. Over the last decade intensive research and development has been carried out in order to develop powerful composites using natural fibres, offering good bio-degradability and sustainability. A biodegradable material will slowly undergo biodegradation by surrounding microorganisms, bacteria, and exposure to the elements and hence could provide solutions to end-of-life issues after service life [1]. Nowadays, the fibres resulting from wood, animals, leaves, grasses and other natural sources are commonly used as reinforcement in composites used for various applications, like automotive (interior and exterior), building, ship, packaging* etc.*, due to their unusual properties compared to other synthetic fibres. Advances in manufacturing techniques in natural fibre-reinforced composites have allowed the car industry to utilise these composites in interior trimmings.

**Figure 1:**
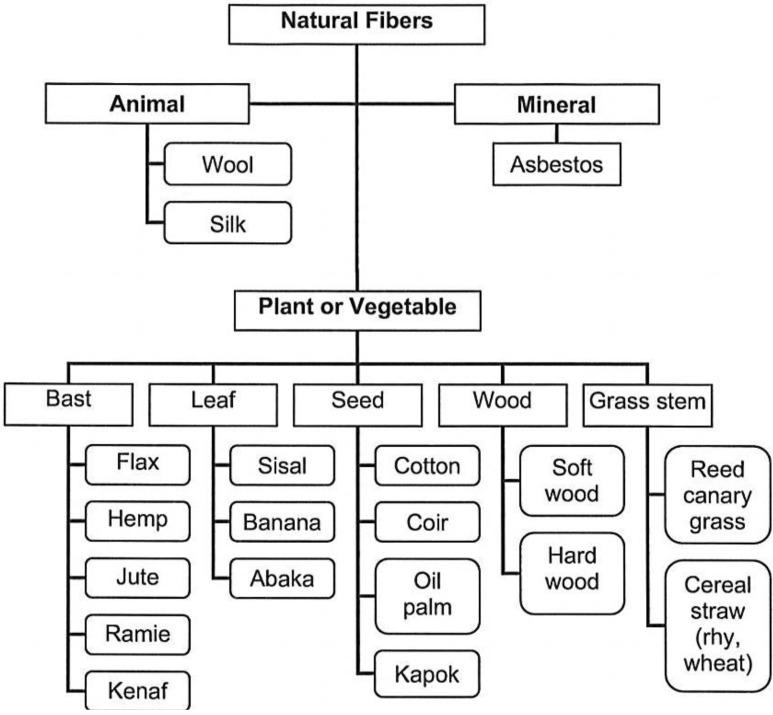
Classification of natural fibres according to origin.

Besides the environmental benefits, compared to glass fibre composites, the natural fibre reinforced composites with the equivalent performances have higher fibre content, resulting in less pollution from synthetic polymer matrix, and much lighter weight, reducing the amount of driving fuel in automotive applications. Table 1 summarises the general reasons for using natural fibres to reinforce polymers. Yu and his co-workers [2], Saheb and Jog [3], Avella [4], and Holbery and Houston [5] have reviewed natural fibre composites with their classifications, properties and potential applications. The natural fibres are divided into the groups of animal (wool and silk), mineral (asbestos) and plant/vegetable (bast, leaf, seed, wood, and grasses) as shown in Figure 1. The development of natural fibre composites is limited due to several issues: (1) the thermal degradation of natural fibres could decrease the mechanical properties (toughness and bending strength), result in poor organoleptic properties (odour and colour) and possible production of volatiles at processing time over 200 °C; (2) the high moisture content of natural fibres, especially cellulosic fibres, could lead to poor dimensional stability and process-ability, and porous issues; (3) the composites exposed outdoors may bio-degrade by ultraviolet light; (4) the dispersion of natural fibres is affected by the strong inter-fibre bonding; and (5) the incompatibility between hydrophobic polymer matrix and hydrophilic natural fibres [3].

**Table 1:** Comparison between natural fibres and synthetic fibre [6,7].

Fibre	Advantages	Disadvantages
Natural fibre	Biodegradable	Inhomogeneous quality
	Low density/price	Dimensional instability
Synthetic fibre	Moisture resistance	Difficult in recycle
	Good mechanical properties	Relative high price

Among the abundance of natural fibres, bast fibres (flax, hemp, jute* etc.*) are commonly used in composite preparation. Summerscales *et al*. [8], Anandjiwala and Blouw [9], and Cao* et al.* [10] have reviewed the research and development of bast fibres, derived from the outer cell layers of the plant stems. Bast fibre stems have a high Young’s modulus up to 140 GPa, comparable to aramid fibres. The typical mechanical properties of the important bast fibres are shown in Table 2. The mechanical merits together with their biodegradability make bast fibres to be increasingly considered as reinforcements for composites in the sustainable future. Due to the environmental benefits and attractive performance of flax fibres from natural sources, the use of flax fibres to reinforce polymeric matrices has been significantly developed for various applications (vehicle, transport, construction* etc.*) in recent years.

**Table 2:** Typical properties of some bast fibres compared to E-glass [11,12].

Fibres	Density (kg/m^3^)	E-modulus (GPa)	Tensile strength (MPa)	Elongation at break (%)
E-glass	2550	71	3400	3.4
Flax	1530	58 ± 15	1339 ± 486	3.27
Hemp	1520	70	920	1.7
Jute	1520	60	860	2
Kenaf	1193	14–38	240–600	–

Many reasons including oil-use capacity, government environment legislation as well as sustainability concern, are responsible for the increased emphasis on flax fibre (one of the bast fibres) to produce composites [13,14]. Additionally, in terms of mechanical performances, flax fibres are perceived to meet high requirements and at the same time have extra advantages (e.g., specific modulus-modulus to weight ratio, safer working conditions, low machine wear, health advantages, recyclability* etc.*) over synthetic fibres [15,16]. The study of flax fibre reinforced bio-composites has been accelerating year by year from the selection of polymeric matrices (thermoplastic, thermoset, bio-matrix) to various treatments for property improvements. Currently no review papers focusing on the flax composites have been published to summarize academic progress in this area. This paper reviews the flax fibres, the fibre treatments and their developed composites based on different matrices to provide basic and concise information for flax composite-related studies in the future.

## 2. Flax Fibres as Reinforcement

### 2.1. Origin of Flax Fibres

**Figure 2:**
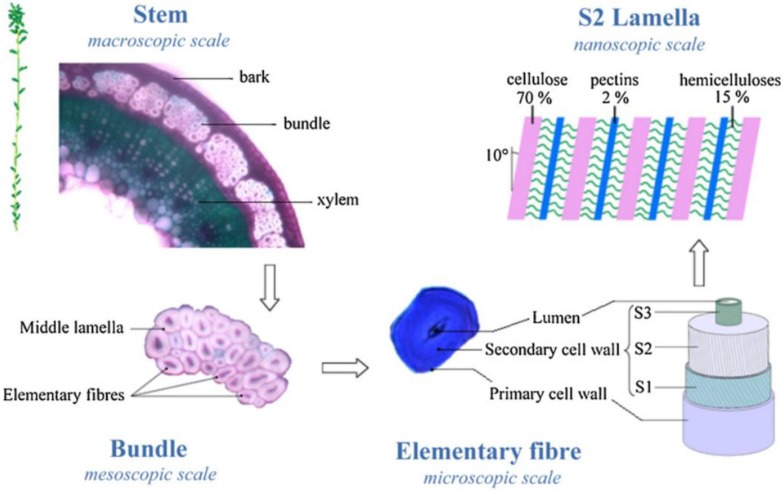
Composition and cross section of flax stems. Adapted with permission from [17]. Copyright 2013 by Elsevier.

Flax fibres come from the flax plant, one species of *Linum usitatissimum* is bred, and is widely cultivated in West Europe where the daily temperature is generally below 30 °C [17]. The flax plant has a life cycle of 90–125 days including vegetative, flowering and maturation periods [17]. The diameter of the flax stem is in the range of 1–2 mm, with a height of about 80 cm. It can be seen from Figure 2 that there are three layers—bark, bundle and xylem—in the flax stem. The outer layer of bark functions as a protective cover from external attacks except for the penetration of water and other nutrients [17].

Figure 2 represents the structure and composition of the origin flax stems. During the fibre processing, the bark, however, together with xylem is eliminated to leave fibre bundles consisting of elementary fibres. Technical fibres are extracted by partially separating the fibre bundles in the flax plant and can be as long as the stem length (approximately 1 m). Unlike the technical fibres, the length of elementary fibres varies between 2 cm and 5 cm, and the diameter is about 19–25 µm. The polyhedron shapes (five to seven sides) help pack the elementary fibres together [18]. The elementary fibres have primary and secondary cell walls, both of which are cellulose material. Cellulose fibrils (diameter between 0.1 µm and 0.3 µm) are embedded in concentric lamella composed of about 2% pectins and 15% hemicellulose which contribute to the thermal degradation and water uptake of the fibres [19]. They can be highly oriented with the fibre axis and thus crystallised in the cell walls to provide high tensile strength [18].

### 2.2. Properties of Flax Fibres

#### 2.2.1. Tensile Properties

**Figure 3:**
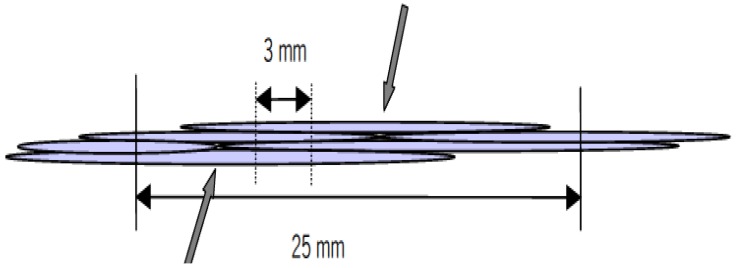
Schematic representation of failure mechanism of flax fibres. At 25 mm clamping length, elementary fibres slip over each other. At 3 mm, cracks run through the cross section of the elementary fibres. Adapted with permission from [16]. Copyright 2013 by Eindhoven University.

It has been reported that there is no large scale plastic deformation of flax fibres in stress-strain behavior as the amorphous regions between fibril are oriented [16]. Baley* et al.* [20] found a longitudinal Young’s modulus of 59 GPa and transverse modulus of approximately 8 GPa. The clamping length of fibres, however, has a great effect on the tensile strength. Bos [16] found that the tensile strength of the technical fibres has a plateau value of 500 MPa, which increases significantly below the clamping length of 25 cm. Two main reasons are mentioned in his report: (1) less critical flaws; (2) changes in failure mechanism. Flaws, such as kink bands resulting from the isolation process* etc.*, are reduced by decreasing fibre length to increase the fibre strength. On the other hand, for a large clamping length, failure takes place through the weak interphase, while the cracks can only propagate through cell walls at a clamping length below the elementary fibre length lying between 20 mm and 50 mm as seen in Figure 3. The mean value of the tensile strength of technical fibres is only 57% of the elementary fibre strength, 1522 ± 440 MPa, due to the bulk effect.

In some areas, flax fibres are competitive to glass fibres, and hence are reasonably acceptable as replacement. Except for the specific properties of flax fibres as shown in Table 3, three other reasons have been stated to make the application of flax fibre more attractive: (1) cheaper than glass fibres; (2) less toxic; (3) high strength to weight ratio. Normally, the flax fibres have a relatively low price compared to glass fibres. In addition, glass fibres are suspected of causing lung cancer, but there is no such problem for natural fibres [16]. The thermal recycling of the flax fibres (burning of flax fibres with few slags left) hasa great advantage over glass fibres.

**Table 3:** Tensile properties of glass and flax fibres [16].

Property	E-glass	Flax fibres
Diameter (µm)	8–14	10–80
Density (g/cm^3^)	2.56	1.4
E-modulus (GPa)	76	50–70
Tensile strength (GPa)	1.4–2.5	0.5–1.5
Specific E-modulus (Gpa/g·cm^−3^)	30	36–50
Specific tensile strength (GPa/g·cm^−3^)	0.5–1	0.4–1.1

#### 2.2.2. Compressive Properties

**Figure 4:**
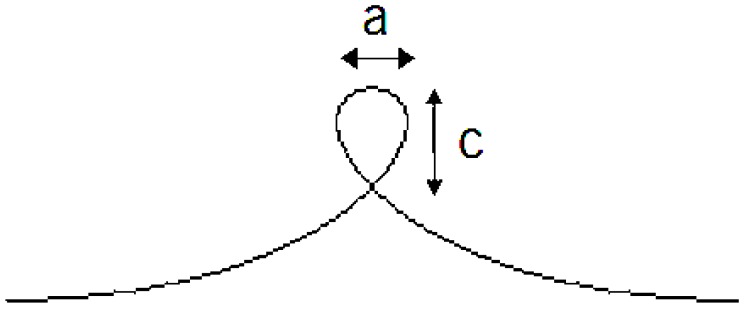
Loop test for compression. The ratio c/a changes at failure point.

Similar to glass fibres, the compressive strength of flax fibres could be measured by the elastic loop test (Figure 4). Flax fibres usually fail in the top of the loop because of the highly oriented structure. The compressive strength, σ_c_, is calculated from [16]:

(1) $$\sigma_{c}=\frac{1.34\;E_{C}\;d}{C_{c}}$$

where the *E*_c_ is the elastic modulus for both tensile and compressive; *d* the fibre diameter; *C*_c_ the point of failure. Fibre samples were tested by Bos [16] and a compressive strength range of 830–1570 MPa was obtained.

#### 2.2.3. Physical Properties

The degradation of flax fibres is a crucial aspect in the development of natural fibre composites and thus has a bearing on the curing temperature in the case of thermosets and extrusion temperature in thermoplastic composites. Cellulose is the main component of natural fibres, and thus controls the major degradation behavior of flax fibres. The degradation routes for cellulose upon heating are discussed in the literature [21]. The glycosyl units are decomposed at low temperature, followed by their depolymerisation at high temperatures. Then the formed substances like levoglucosan decompose into gas at higher temperatures.

The effect of thermal degradation on mechanical properties of flax fibres was investigated by Gassan and Bledzki [22]. They placed the untreated flax fibres in a laboratory oven between 170–210 °C for a maximum of 120 min. Then the tenacity of flax fibres was measured by a tensile test. The tenacity decreased slightly below 170 °C, whereas there was a rapid decrease of tenacity and polymerisation degree at temperatures above 170 °C. The behavior strongly depends on the exposure time and temperature. Additionally, a slight increase of crystallinity was observed after heat treatment, as a result of chain scissions.

Stamboulis, Baillie and Peijs [23] demonstrated that moisture absorption varied between Duralin flax fibres and Green flax fibres. Duralin fibres absorbed less water and retained a smoother fibre surface after fibre separation than Green flax fibres. The tensile strength of Duralin flax fibres increased to a maximum tensile strength at a humidity of 66% and decreased afterwards. The reason is that water plasticised the fibres at low absorbed water content but this effect was less important at large moisture content.

### 2.3. Development of Surface Treatment on Flax Fibres

To improve the adhesion between hydrophilic flax and hydrophobic polymer matrix, many studies on using chemical or physical treatments have been published [24,25,26,27]. Some typical treatment routes from published articles are collected in Table 4. Common treatments include mercerization, dewaxing, silane treatment, acrylation, peroxide treatment, coatings, and impregnation with a dilute epoxy [28,29,30].

**Table 4:** Different treatments of flax-reinforced composites.

Fibre/matrix	Chemical treatment	Conditions	Effect on properties	Reference
flax/PP	esterification	10 wt % MA, 25h, 50 °C	highest flexural and tensile strength	[26]
flax/phenolic	esterification	25 wt % MMA, 30min, 210 W	more moisture retardant	[27]
flax/epoxy	alkali treatment	5 wt % NaOH, 30 min	tensile strength 21.9%; flexural strength 16.1%	[31]
flax/epoxy	alkali treatment	4 wt % NaOH, 45 s	transvers strength, 30% increment	[32]
flax/polyester	silane treatment	0.05 wt %, 24 h	hydric fibre/matrix interface	[33]
flax/pp	esterification	MA-PP coupling agent	interphase compatibility	[34]

*MMA—methylmethacrylate; MA—maleic-anhydride.

#### 2.3.1. Silane Treatment

**Figure 5:**
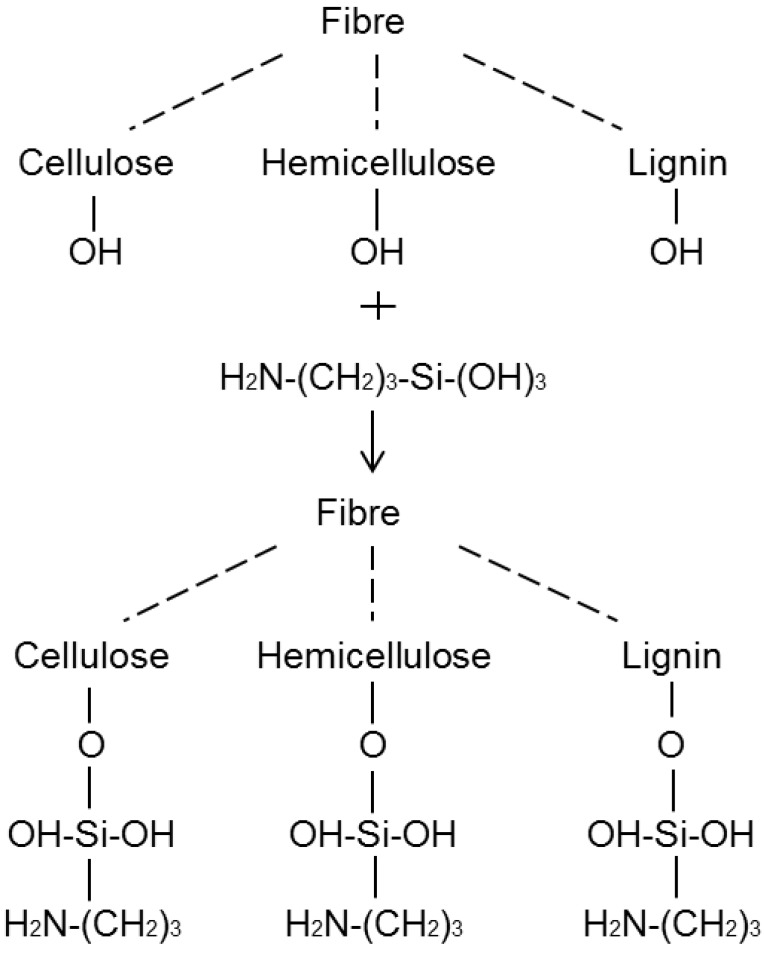
Grafting of silanols on flax fibre surface (redrawn from Singha *et al*. [35]).

Coupling agents usually improve the degree of crosslinking in the interface region and offer a perfect bonding. Among the various coupling agents, silane coupling agents were found to be effective in modifying the natural fibre-matrix interface. Proper treatment of fibres with silane can increase the interfacial adhesion to the target polymer matrices and improve the mechanical performances of the resulting fibre/polymer composites. Silane is hydrolyzed forming reactive silanols and is then adsorbed and condensed on the fibre surface (sol-gel process). The hydrogen bonds formed between the adsorbed silanols and hydroxyl groups of natural fibres may be further converted into covalent bonds by heating the treated fibres at a high temperature (see Figure 5).

Xie and his co-workers [13] reviewed silane coupling agent modification to natural fibre composites and found out improvements in strength, moisture absorption and fungal resistance for UP and epoxy composites. The suitable silane modification for fibres in epoxy composites is aminopropyl triethoxy siloxane (APS) and for methacryloxypropyl trimethoxysilane (MPS). APS solution (3%) combined with alkali treatment was found to provide better moisture resistance [35].

#### 2.3.2. Acetylation

Acetylation is a well-known esterification method originally applied to wood cellulose to stabilize the cell walls against moisture, improving dimensional stability and environmental degradation. In lignocellulosic material the acetic anhydride reacts with more reactive hydroxyl groups (OH) in lignin and hemicellulose (amorphous material), whereas the hydroxyl groups of cellulose (crystalline material) prevent the diffusion of the reagent and result in a low extent of reaction [36]. Tensile and flexural strengths of flax/PP composites were found to increase with increasing degree of acetylation up to 18% [37].

#### 2.3.3. Anhydride Treatment

**Scheme 1:**
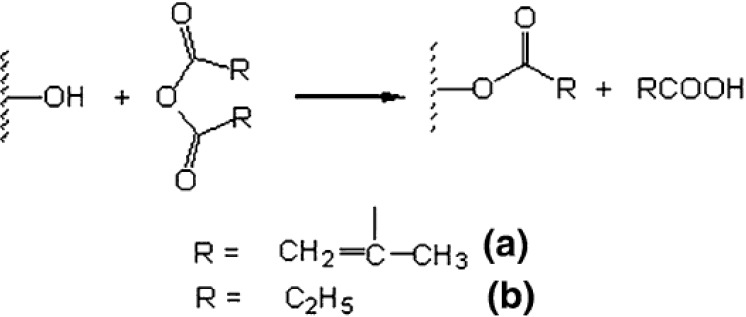
Schematic presentation of the reaction between –OH groups of flax fibres and (**a**) methacrylic; (**b**) propionic anhydrides.

Anhydride treatment is usually carried out by utilizing maleic anhydride or maleated polypropylene (PP) or polyethylene (PE) in a toluene or xylene solution, where the fibres are immersed for impregnation and reaction with the hydroxyl groups on the fibre surface. Hugues, Carpenter and Hill [24] reported that methacrylic and propionic anhydride modification showed a marked effect on yield properties (yield point, on set of yield* etc.*) of flax composites. There are two modification mechanisms used: (a) reactive vinylic group introduced by methacrylic anhydride (MA); and (b) hydrocarbon coating on the surface to improve the hydrophobicity by propionic anhydrides (PA) (see in Scheme 1). The trend of bond strength was observed as unmodified < PA < MA, and was related to the work of fracture trend: MA < PA < unmodified due to the fact that the debonding behavior was inhibited by good bonding strength. The treatment of natural fibres with MAPP copolymer decreased the polar component of the surface energy to a similar value of PP and hence improved the wettability [26].

#### 2.3.4. Mercerization/Alkali Treatment

Alkali treatment of natural fibres, also called mercerization, is the common method to produce high-quality fibres. Mercerization has an effect on the chemical composition of the flax fibres, degree of polymerization and molecular orientation of the cellulose crystallites due to cementing substances like lignin and hemicellulose which are removed during the mercerization process [34]. Alkali treatment also converted the crystalline form of cellulose I into cellulose II [32]. The extent of this transformation could be to some extent reflected by the intensity ratio of the stretching modes of symmetric (C–O–C) and asymmetric (C–O–C) through FT Raman spectroscopy [25]. Mechanical properties of polystyrene composites reinforced with chemically treated flax fibre were investigated and it was observed that mercerization of flax fibres improved the mechanical properties of polystyrene composites [38].

The changes in surface morphology and chemical compositions, along with mercerization greatly influence the thermal degradation of flax fibres and hence the processing temperatures. Bledzki* et al.* [39] reported that the degradation temperature of flax fibres increased from 319 °C to 360 °C after acetylation (34% acetylation). It was also stated that the thermal stability of flax fibres increased after alkali treatment due to the composition change on the removal of lignin and hemicellulose [40].

#### 2.3.5. Enzymatic Treatments

Enzymes are an increasingly interesting option as such or when combined with chemical and mechanical methods for modification and processing of biomaterials. This is due to the fact that enzymes are highly specific and efficient catalysts and they work in mild, energy-saving conditions. Oxidative enzymes, such as laccases or peroxidases, can be used to activate and further functionalise lignocellulosics [41]. The primary reaction of laccase is the oxidation of phenolic hydroxyls to phenoxy radicals in the presence of oxygen. Laccases can thus be used to activate lignin, lignans, and different types of lipophilic extractives present in the complex lignocellulosic materials [42]. It was found that the lignin content of single cellulose fibres decreased from 35% to 24% with laccase treatment.

Research activities in the use of laccase in various application areas have recently been reviewed by Kudanga* et al.* [43]. The laccase-catalysed modification can be used to tailor the properties of various lignocellulosic materials, including flax fibre materials based on the application needs [44]. Natural phenols, such as syringaldehyde, acetosyringone and p-coumaric acid, in combination with laccase treatment, were recently developed to graft on the flax fibres and offered antimicrobial properties [45,46]. Lauryl gallate (LG), a hydrophobic compound with the strongest internal sizing effect, was grafted onto cellulosic fibres, and the results showed a significant reduction in water penetration [47,48].

#### 2.3.6. Other Treatments

There are still a number of available pre-treatments, such as benzoylation [49], etherification [50], isocyanate treatment [51], peroxide treatment [38], sodium chlorite [52] and stearic acid treatment [53]. In benzoylation treatment, benzoyl chloride is most often used in fibre pretreatment. The inclusion of a benzoyl group in the fibre is responsible for the decreased hydrophilic nature of the treated fibre, decreasing its water absorption, but also increasing its strength properties. For the modification of cellulosic fibres by etherification sodium hydroxide plays an important role in forming a charged intermediate species with the fibre, which allows faster nucleophilic addition of epoxides, alkyl halides, benzyl chloride, acrylonitrile, and formaldehyde. The isocyanate group can react with the hydroxyl groups on the fibre surface forming covalent bonds, thus improving the interface adhesion. Organic peroxides tend to decompose easily to free radicals (RO.), which further react with the hydrogen group of the matrix and cellulose fibres. Sodium chlorite (NaClO_2_) is used usually in bleaching fibres; however, it could delignify lignocellulosics. Fibre treatment of stearic acid (CH_3_(CH_2_)_16_COOH) in ethyl alcohol solution was reported in that this treatment removed non-crystalline constituents of the fibres, thus altering the fibre surface topography. It could be interesting to add a conclusion: these treatments are not very eco-friendly and the better way to use natural fibres is to use them as received without chemical treatments.

## 3. Properties and Characterization of Flax Polymeric Composites

Like common polymer composites, either thermoplastics or thermosets could be used with flax fibres and offer various mechanical properties. From the matrix point of view, thermoplastic matrices like PP and PE are ductile, easy to process and simple to recycle. On the other hand, processing temperature and time must be properly controlled so as to decrease the viscosity for suitable wetting and not degrade the fibres. The final cross-linked thermoset structure of low viscosity of the monomers highly increased the mechanical and thermal properties. However processing is more demanding and recycling is restricted. Concerning the increasing needs of recyclable materials, bio-degradable polymeric matrices (polylactic acid (PLA), soy protein epoxy and tannin phenolic resin) have attracted more and more attention from academics and industrialists. Table 5 shows the collected mechanical properties of flax-reinforced composites based on different matrices. Except for mechanical performance, the physical information of flax fibres and associated composites, could be obtained through various approaches, such as DSC (differential scanning calorimetry) [54], DMA (dynamic mechanical analysis), TGA (thermogravimetric analysis) [54], X-ray diffraction [34], SEM (scanning electron microscopy) [54,55] and FTIR (Fourier transform infrared spectroscopy) [53,56].

### 3.1. Thermoplastic Polypropylene Based Flax Composites

Many studies [37,57,58,59,60] have concentrated on flax/thermoplastic composites and have provided valuable information. From the investigations of Van de Velde and Kiekens [37], PP is the most suitable thermoplastic matrix for flax-reinforced composites due to its various advantages, such as low density, low thermal expansion, good resistance to water and recyclability. The adhesion between hydrophilic flax fibres and hydrophobic PP is the problem of most concern, modification technologies therefore have been applied to improve it. Boiling of flax and use of chemicals (e.g., maletic acid) was proven to be good for adhesion modification in order to increase mechanical properties [61,62,63]. Garkhail, Heijenrath and Peijs [64] prepared flax/maletic-anhydride grafted PP (MA-PP) composites by two production methods, called film-stacking and paper-making process. The effect of fibre length on composite stiffness and tensile strength showed very little agreement with the model predictions shared with other scientists [65,66]. The critical length was thought to be reduced by the addition of MA-PP, compared to PP/flax. Fibre volume however had a significant effect on the final mechanical properties of composites. The influence of physical structure of flax fibres on mechanical properties was investigated by Van Den Oever, Bos and Van Kemenade [67]. 40 vol % hackled and 40 vol % scotched fibre/PP were used to compare with the theoretical predictions. The results indicated that combing the flax fibres (hackled fibres) removed some weak lateral bonds with detriment to tensile and flexure strength. The compressive behavior of composites is also related to the presence of kink bands which can be removed by combing [68].

**Figure 6:**
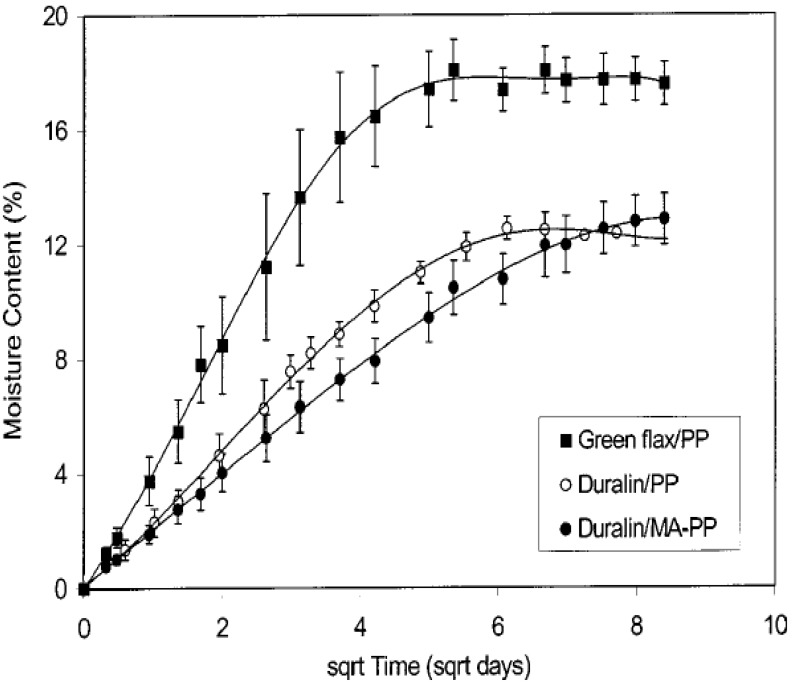
Moisture content of flax/PP composites as a function of time. Adapted with permission from [69]. Copyright 2013 by Springer.

**Figure 7:**
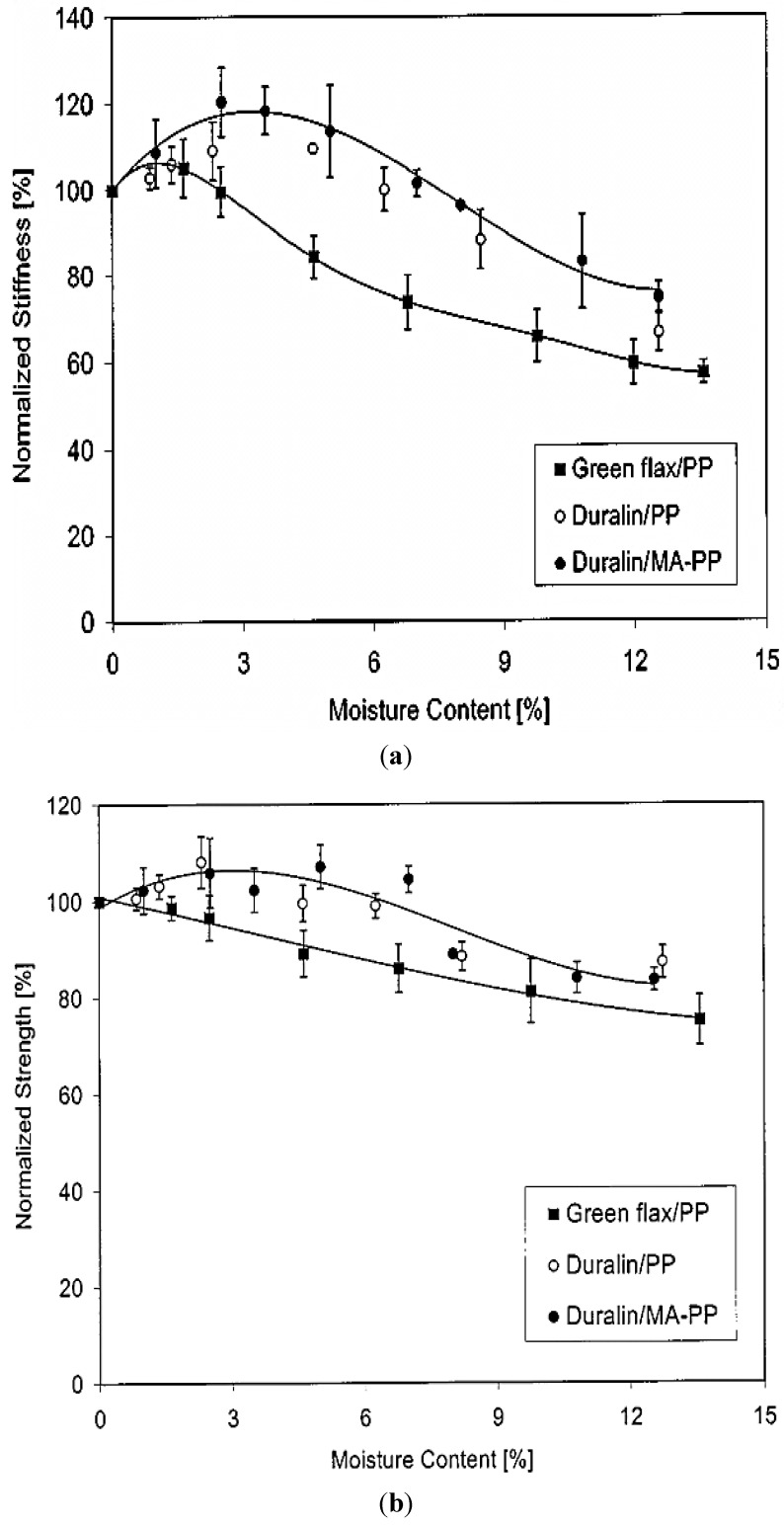
Moisture content* versus* mechanical properties: (**a**) stiffness; (**b**) strength Adapted with permission from [69]. Copyright 2013 by Springer.

The hydrophilic character of bio-composites cannot be neglected, resulting from which water absorption/ageing plays an important role in degradation and decrease of mechanical properties. Recent studies [23,69,70,71] have reported that the effect of water uptake in bio-composites limits their outdoor applications. In general, there are three ways to understand the term “water absorption”: (1) water diffuses directly into the matrix; (2) through interphase matrix/reinforcements; and (3) by imperfections, like pores and cracks. A study of moisture absorption and environmental durability of flax (Green and Duralin)/PP composites was conducted by Stamboulis and his co-workers [69]. At room temperature, composites absorbed water towards an equilibrium and then became time-independent. The plotted curve of moisture content* versus* root time (Figure 6) could be explained by Fick’s law. Green flax/PP composites were clearly more sensitive to water than Durbin flax ones, meanwhile the addition of MA (maleic-anhydride)-PP lowered the initial water uptake rate with little effect on the maximum moisture content. In terms of mechanical properties, the moisture content affected stiffness of flax/PP composites more than tensile strength (Figure 7). The stiffness increased somehow at low moisture content due to the filled interfacial gap by swelling flax fibres, while it decreased significantly at 7% moisture content. It is more likely because of fibre degradation caused by fungus development, not a micro cracking mechanism in a brittle matrix.

**Table 5:** Testing data of mechanical properties of flax reinforced composites.

Fibre/matrix	Processing method	Tensile strength (MPa)	Tensile modulus (GPa)	Flexural strength (MPa)	Impact strength (kJ/m^2^)	Reference
flax/bio-thermoset(MSO)	compression moulding	≈ 50–120	≈ 6–15	180 (max)	–	[72]
flax/bio-thermoset (MMSO)	compression moulding	≈ 50–120	≈ 7–15	201 (max)	–	[72]
Arctic Flax/ Epoxy(50:50)	resin transfer moulding	280	40	–	–	[73]
plain woven flax/epoxy	hand lay-up	–	–	≈ 78–100 (MPa/g cm^−3^)	≈ 17–35 (kJ/m^2^/g cm^−3^)	[74]
plain-woven flax/thermoset	compression moulding	280	32	250	15 (Charpy)	[75]
flax yarn/SPC resins	pultrusion	298	4.3	117	–	[19]
flax/Lactic acid resins(70:30)	compression moulding	62	9	96	–	[76]
flax/PLA	injection moulding	≈ 40–55	≈ 3–6	–	≈ 9–11 (Charpy)	[77]

### 3.2. Thermoset-Epoxy Resin Based Flax Composites

The mechanical performances of epoxy based flax composites have been widely investigated [73,78,79,80]. Hughes* et al.* [24] produced unidirectional flax/epoxy composites and directed their investigation towards tensile deformation behavior. The work revealed the non-linear stress-strain relationship under tensile loading with respect to the flax/matrix adhesion and the presence of kink bands. Muralidar [81,82] studied epoxy composite reinforced by flax in the form of hybrid performance (plain weave fabric and rib knitted structure) through a lay-up method. He pointed out that the compressive properties were mainly contributed by the matrix whereas the tensile properties of woven composites were highly influenced by the flax volume fraction in the tension direction. The effect of woven flax fabric on fracture toughness of flax/epoxy composites was reported by Liu and Hughes [83]. The well-packed fibres in the textile lead to a high fibre volume fraction and hence the improvement of fracture toughness up to 9 MPa∙m^1/2^ compared to pure resin (about 1.8 MPa∙m^1/2^). Additionally, the results strongly depended on the testing directions (weft and warp) with different fibre densities. Oksman [73] found that Arctic Flax /epoxy composites had outstanding mechanical properties (e.g., maximum tensile strength of 280 MPa) and presented a better specific modulus of 29 GPa/g∙cm^−3^ than that of glass/epoxy composites. Liang and his co-workers [78] compared fatigue behaviours between glass fibre/epoxy and flax fibre/epoxy composites. They reported that flax composites had the advantages of a relatively stable modulus under cycling load over glass fibre-reinforced composites.

**Figure 8:**
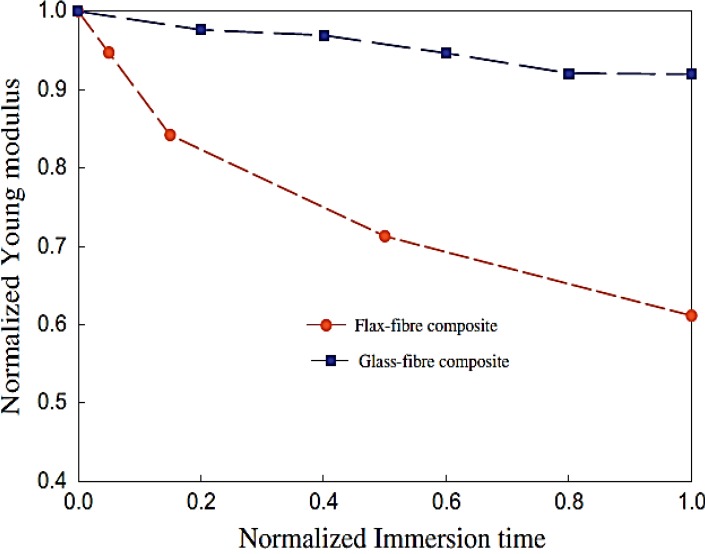
Normalised Young’s modulus (modulus/modulus at time = 0)* versus* normalised immersion time (test time/saturation time). Adapted with permission from [70]. Copyright 2013 by Elsevier.

**Figure 9:**
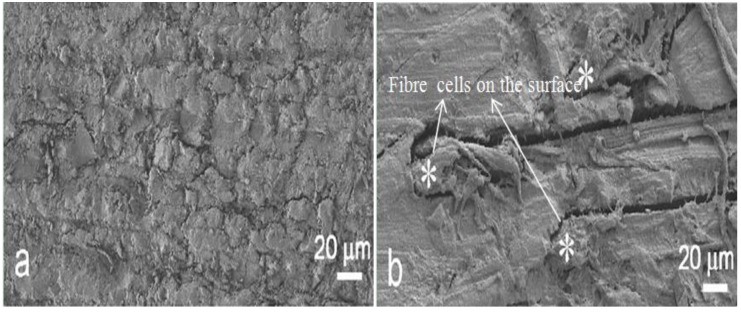
Scanning electron microscopy (SEM) images of flax/epoxy composites: (**a**) origin specimen; and (**b**) after eight wet-dry cycles. Adapted with permission from [84]. Copyright 2013 by Elsevier.

Like all the natural fibre composites, environmental durability plays a very important role in flax/epoxy composites. Assarar and his co-workers [70] reported the influence of water on the ageing properties of epoxy composites with 11 flax unidirectional plies. The failure stress decreased around 13% after the first 1-day water immersion. Then the reduction of failure stress was only an extra 2% between 1 day and 20 days of immersion. From Figure 8, a 30% decrease of normalised Young’s modulus of flax/epoxy in the first 10 days showed a much worse result from water ageing than glass fibre composites. Hewman [84] tested the water damage of plain-weave flax/epoxy composites (eight plies) through the wet-dry cycle procedure. The tensile strength after the first wet-dry cycle was found to be 89% of the unconditioned value, and dramatically dropped to 16% after the fourth cycle. Penetrated water expanded the flax fibres and resulted in matrix/technical fibre gaps (SEM images in Figure 9) after drying due to the different shrinkage degrees of fibres and matrix. The increased number of wet-dry cycles enlarged these gaps (auto-accelerative process) and consequently weakened the load transfer.

### 3.3. Bio-Degradable Poly(Lactic Acid) (PLA) Based Flax Composites

The natural fibre (e.g., hemp, flax, jute and sisal) reinforced composites to replace glass-reinforced composites have been well-established for several years because of increasing environmental awareness. Nevertheless, due to the petroleum-based polymer matrices (e.g., PP and epoxy), the composites are not fully degradable, leading to difficulty in the recycling process. Hence, a new generation of fully bio-materials needs to be developed.

**Figure 10:**
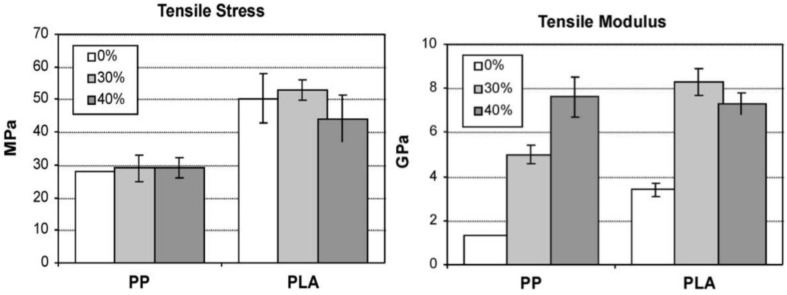
Tensile properties of flax/PLA compared to flax/PP composites. Adapted with permission from [87]. Copyright 2013 by Elsevier.

To improve the sustainability and eco-efficiency, bio-degradable poly(lactic acid) has been increasingly used together with flax fibres for fully bio-composites [4,85,86,87]. PLA can be derived from corn starch, sugarcane* etc.*, while it is also able to be synthesized, especially for industrial production. Akesson* et al.* [76] produced and studied fully biodegradable composites from PLA and flax fibres. The mechanical properties of final composites were increased by varying the fibre content from 40 wt % to 70 wt %, but drastically reduced with a fibre ratio of 75 wt %. The storage modulus of PLA composites with 70 wt % flax fibres was 9.32 GPa and 3.29 GPa at 20 °C and 140 °C, respectively. The humidity ageing tests showed that the tensile modulus was reduced from 9 GPa to 2.5 GPa after 1000 h exposure time at 95% humidity and 38 °C, meanwhile the tensile strength was reduced by about 70%. It was demonstrated by Bax and Müssig [77] that the flax/ PLA composites had a higher Young’s modulus of 6.31 GPa than Cordenka/PLA composites. The impact strength increased to 11 kJ/m^2^ with a fibre mass fraction of 30 wt %. Oksman* et al.* [87] manufactured flax (long heckled fibres)/PLA composites by compression moulding and then compared them to the commercial flax/PP composites used for automotive panels. The composites with 40% flax content showed good fibre dispersion and over 50% higher tensile modulus up to 7.3 GPa than flax/PP composites (as seen in Figure 10). The DMA results indicated that the reinforcement of flax fibres increased *T*_g_ from 50 °C for pure PLA to 60 °C for the composite, and presented a cold crystallization at 80 °C. For the adhesion bonding measurement of flax/PLA composites, Le Duigou and his co-workers [88] designed a microbond test to estimate the interfacial shear strength. With decreasing thermal treatment rate, the shear strength increased from 33 MPa for 93 °C/min to 38 MPa for 1.5 °C/min, as a result of the thermal residual stress from the crystallization.

### 3.4. Bio-Epoxy Resin Based Flax Composites

Vegetable oil-derived renewable epoxy resin is a potential substitute of petroleum-based resin for flax composites to be used in automotive and construction applications. Bio-epoxy resins have been developed with flax fibre reinforcement [72,75,89,90,91]. Flax yarn and flax woven fabric reinforced soy protein concentrated resins (SPC) were prepared by Huang and Netravali [19] for the comparison of tensile and flexural properties. Two layers of unidirectional flax yarns were impregnated with resin solution by winding fabrication, and then air cured at 35 °C for 24 hours, followed by a 25 minute hot pressing at 120 °C of 8 MPa. Flax fabric composites were made of four resin-coated flax fabrics cured at the same hot pressing condition. Flax yarn composites showed the highest tensile strength of 298 MPa and flexural strength of 117 MPa, while for flax fabric composites, failure stresses of 62 MPa and 83 MPa were observed in the warp and weft direction, respectively. The reinforcement of both flax yarns and flax fabrics results in high failure strain and high toughness.

Adekunle* et al.* [72] prepared bio-epoxy composites reinforced by hybrid non-woven and woven flax fabrics. MMSO (methyacrylic anhydride modified soybean oil) concentrate bio-epoxy resins and MSO (methacrylated soybean oil) resin were applied. The composite laminates consisted of three non-woven flax mats sandwiched between four woven fabrics (0°, 45°, 90° orientation) with different stacking sequences. The fibre configuration of [04/N3] results in the composite tensile strength up to 119 MPa and modulus up to14 GPa. A flexural strength of 201 MPa and modules of 24 GPa were also achieved. MMSO offered additional methacrylate function groups, and hence the final composites exhibited better mechanical properties with higher level of cross-linking. The extra addition of styrene in the bio-thermosets improved the mechanical (e.g., brittle) properties due to the low viscosity of molten styrene and resulted in a better fibre/matrix adhesion.

**Figure 11:**
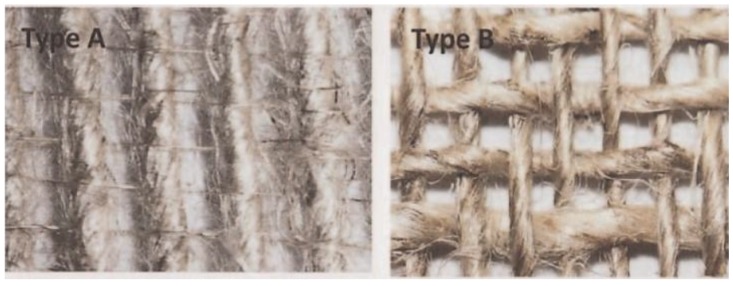

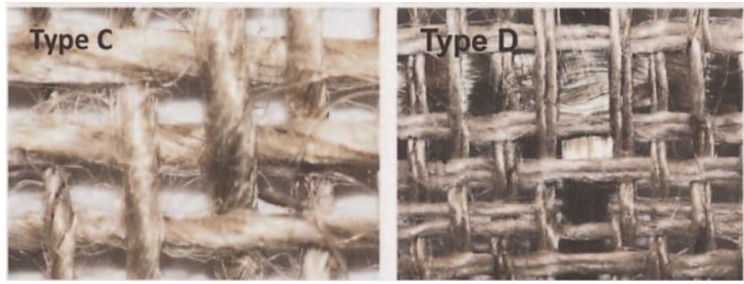
Weave architecture types: (**A**) plain; (**B**,**C**) two different twill; and (**D**) dobby [75]. Adapted with permission from [17]. Copyright 2013 by SAGE.

A study of the influences of weave architectures on the mechanical properties of flax fibre/bio-epoxy composites was conducted by Adekunle *et al.* [75]. The woven fabrics were in the forms of plain, twill and dobby as shown in Figure 11. The tensile, flexural and impact properties were found to follow the trend: Plain weave type < twill type < dobb type. The inherent thin weft yarns in plain weave fabric were neglected, and hence the almost unidirectional properties along the warp yarn. The *T*_g_ of flax/bio-epoxy composites was around 85 °C from the tanα plot, which was a little higher than *T*_g_ of 70 °C from the loss modulus data.

### 3.5. Bio-Phenolic (Tannin) RESIN Based Flax Composites

**Figure 12:**
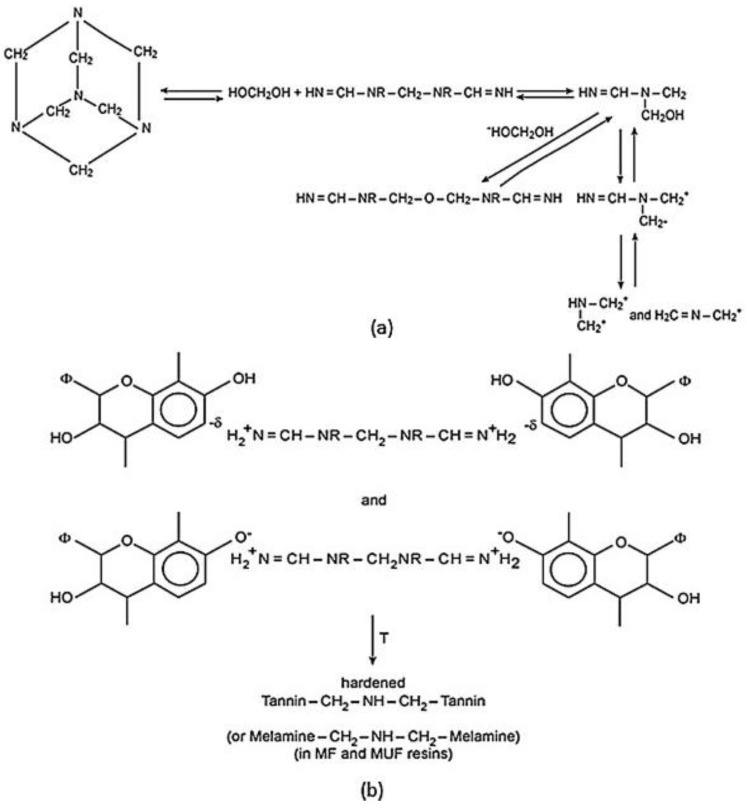
The chemical reaction between tannin and hexamine: (**a**) decomposition of hexamine; and (**b**) polycondensation of condensed tannin [98].

Tannin resin, a natural phenolic resin, has been reinforced by flax fibres to offer environmental benefits and desirable characteristics aimed at reducing the environmental footprint of superlight electric vehicle applications such as vehicle body panels, crash elements, side panels and body trims [92,93,94]. Tannin resins can be obtained from large varieties of plants (e.g., wattle, myrtle, pine* etc.*) through the extraction with water and can be formed by crosslinking with formaldehyde and hexamine (Figure 12) [95]. The use of tannin as matrix has been paid more and more attention for the following reasons: (i) the non-toxic nature of tannin and related hardeners; (ii) the wide availability of tannin and resulting cheaper cost; and (iii) the fast curing rate of tannin resins. Pizzi* et al.* [96] firstly manufactured the mimosa tannin-based composites reinforced by a non-woven mat of flax fibres and also studied their mechanical properties. There were two natural matrices used: (1) mimosa tannin with 5% hexamine as hardener; (2) mixed tannin/ lignin resins in 50/50 solid content. The low density (8 mm thickness) and high density (1.2 mm thickness) composites were prepared, and followed by tensile tests, three point bending tests and Brinell hardness tests. The tensile strength was largely dependent on the variations of density. The low density composites had a 50% increase of tensile strength from 536 kg/m^3^ to 727 kg/m^3^. By placing several layers together to obtain the required thickness, weak interfacial planes were unavoidable causing delamination rather easily.

Zhu* et al.* [94,97] studied flax/tannin composites with four fibre configurations of nonwoven mats and woven fabric lay-up angles ([0°]_8_, [0°, 90°]_2_ and [0°, +45°, 90°, −45°]_2_). A compression moulding process was conducted to manufacture 50 wt % flax containing tannin-based composites. The longitudinal tensile strength and Young’s modulus went up to 140 MPa and 9.6 GPa, respectively, at the fibre form of [0°]_8_. The relatively good fibre distribution in all directions of [0°, +45°, 90°, −45°]_2_ composites gives rise to the best impact performance. The SEM image of the tensile fractured surface indicated poor flax/matrix adhesion in all woven flax composites, compared to nonwoven composites. The *T*_g_ of around 60 °C was observed for all the composite laminates. Thus there is a need to tailor the fabric arrangement appropriately and to further enhance interfacial adhesion.

### 3.6. Flax Composites Based on Other Matrices

In addition to PP, epoxy, PLA, bio-epoxy and tannin resin, a few other polymeric matrices for flax composites are also available. Saiah and his co-workers [99] fabricated and characterised flax-reinforced composites based on thermoplastics from wheat flour. The stress at failure increased from 4.4 MPa at fibre content with 5 wt % to 8.9 MPa at 20 wt %, while there was an increment of 270% in tensile modulus. They used X-ray diffraction to analyze the crystallinity of the flax fibre. The increase in fibre content leads to an increase in intensity of peaks at 2θ of 15.1°, 16.8°, 22.7° and 34.4°, corresponding to the crystalline structure of flax fibres. In terms of thermal degradation, an observed mass loss peak in the 300–360 °C range of the composites was found to increase with increasing fibre content. Andersons and Joffe [100] pointed out that the experimental tensile strength of a vinylester resin/flax composite can reach the theoretically predicted values only at low fibre volume fraction (up to 20%) due to the somewhat misoreinted fibres caused by heterogeneous and short fibre length.

## 4. Nanotechnology Applied in Flax Composites

Nano-level technology has also been developing at a high speed in flax fibre polymer composites. The recent research activities and developments of nano-level cellulose fibre reinforced composites were reviewed by Eichhorn* et al.* [101]. With respect to the production of cellulose nanofibres and their composites, the review pointed out some issues that are worth noting: (1) structure damage may occur during extraction of nanofibres; (2) lots of energy is required by mechanical ways to separate fibres; and (3) the dispersion of nanofibres is crucial to control the composite quality and can be greatly enhanced by layer-by-layer deposition. Due to the high surface/volume ratio of cellulose nanowhiskers, not only is a high efficiency of stress transfer reached but also chemical modification like grafting of DNA and coupling of chromophores can be applied for various purposes.

Flax bast fibres are one of the major sources to produce cellulose nanofibres, showing great potential for composite applications. Bhantnagar and Sain [102] investigated the flax fibre-derived cellulose nanofibres with diameter range between 10 nm and 60 nm and prepared 10% nanofibre containing polyvinyl alcohol (PVA) composite films. The strong orientation of flax nanofibres gives rise to the high crystallinity of 59% obtained from the X-ray diffractograms. The tensile modulus increased from 2.29 GPa of pure PVA to 6.1 GPa of PVA composites using 10% flax nanofibres. The glass transition temperature of flax nanofibre/PVA composites was found to shift up to 58 °C with increasing fibre content from 5% to 40% [103]. Qua and his co-workers [104] found three degradation steps of nanofibre/PVA composite films by tracking the weight change in TGA curves. The second degradation peak corresponding to the dehydration of PVA, was significantly increased by the addition of nanofibres due to the difficulty in breaking down the strong hydrogen bonding between PVA matrix and fibres.

Except for the nanofibre reinforcement, Huang and Netravali [19] added nano-clay particles to SPC (soy protein concentrated) composites reinforced by flax yarns and flax fabrics separately. The presence of nano-clay particles enhanced composite stiffness (e.g., tensile modulus and flexural modulus), but reduced the failure strain. The reduction of maximum strain may be due to the increased rigidity of the polymer chain and defects caused by clay particles. The highest tensile strength and flexural strength of flax/SPC composites is 298 MPa and 117 MPa, respectively.

## 5. Conclusions

Polymer composites reinforced by flax fibres have attracted much attention from manufacturers and scientists due to the increased environmental awareness. Flax fibres with good mechanical properties (e.g., high tensile strength up to 1000 MPa) and physical properties have been reported as reinforcement for composites used for non-structural and structural applications. It is inevitable that the inherent detriments of flax, including moisture adsorption and incompatibility with some polymeric systems due to high hydrophilicity, present many challenges with respect to composite design and applications. Various chemical treatments, such as mercerization, silane treatment and benzoylation* etc.*, of flax fibres can improve the interface between fibres and matrix.

Flax fibre composites have a wide range of properties, depending on the matrix type, such as thermoplastic, thermosets and biomaterials. Flax/PP composite is the most commonly studied composite and has been commercially used in automotive applications (e.g., vehicle panels). Anhydride treatment is a very efficient way to improve the flax/PP adhesion and hence the mechanical properties. PLA reinforced with flax gives fully biodegradable composites, ideal for replacement of flax/PP. The humidity sensitivity is still a problem as it decreases the long-term material properties. The properties of flax/epoxy composites are strongly influenced by the processing methods and fibre configurations. Resin transfer moulding and compression moulding are preferred for high performance flax/epoxy composites. The bio-epoxy investigated in flax composites is mainly from soybean oil and the final composites have similar independent factors to flax/epoxy composites. Mechanical performance under similar conditions could follow the fibre form trend: plain weave type < twill type < dobb type. Flax/tannin composites have been studied only in the recent 2–3 years, mostly for automotive applications. The ease of processing, good mechanical properties and low overall cost have spurred on further development of flax/tannin composites. Also, the use of nanotechnology (flax nanofibres and the addition of nanoclays in flax composites) highly improves the mechanical performances.
